# Different Forms of Plasticity Interact in Adult Humans

**DOI:** 10.1523/ENEURO.0204-22.2023

**Published:** 2023-07-10

**Authors:** İzel D. Sarı, Claudia Lunghi

**Affiliations:** Laboratoire des Systèmes Perceptifs, Département d’Études Cognitives, École Normale Supérieure, Paris Sciences et Lettres University, Centre National de la Recherche Scientifique, Paris 75005, France

**Keywords:** binocular rivalry, homeostatic plasticity, monocular deprivation, motor learning, neuroplasticity, ocular dominance

## Abstract

Neuroplasticity is maximal during development and declines in adulthood, especially for sensory cortices. On the other hand, the motor and prefrontal cortices retain plasticity throughout the lifespan. This difference has led to a modular view of plasticity in which different brain regions have their own plasticity mechanisms that do not depend or translate on others. Recent evidence shows that visual and motor plasticity share common neural mechanisms (e.g., GABAergic inhibition), indicating a possible link between these different forms of plasticity, however, the interaction between visual and motor plasticity has never been tested directly. Here, we show that when visual and motor plasticity are elicited at the same time in adult humans, visual plasticity is impaired, while motor plasticity is spared. Moreover, simultaneous activation of working memory and visual plasticity also leads to impairment in visual plasticity. These unilateral interactions between visual, working memory, and motor plasticity demonstrate a clear link between these three forms of plasticity. We conclude that local neuroplasticity in separate systems might be regulated globally, to preserve overall homeostasis in the brain.

## Significance Statement

Here, we investigated for the first time the interaction between visual plasticity, motor plasticity, and working memory in a group of adult human volunteers. We found a unilateral interaction between these forms of plasticity: when homeostatic visual plasticity is induced at the same time as motor plasticity or working memory, visual plasticity is disrupted. Our results indicate the existence of a global regulation mechanism in the brain linking different forms of plasticity. Understanding the link between different forms of plasticity is crucial for the development of new neuro-rehabilitative interventions on neurologic diseases and brain injury.

## Introduction

Neuroplasticity in the sensory brain is at its peak during the critical period in development and steeply decreases afterward (Berardi et al., 2000; Pascual-Leone et al., 2005). The visual cortex is one of the most widely used models to study neuroplasticity, with the gold standard paradigm of ocular dominance (OD) plasticity: during the critical period, monocular deprivation (MD) shifts neural responses in the primary visual cortex in favor of the open eye and leaves the deprived eye amblyopic (Wiesel and Hubel, 1963a; Hubel and Wiesel, 1970; Hubel et al., 1977; Drager, 1978; Maffei et al., 1992; Fagiolini et al., 1994). After the closure of the critical period, this OD change is no longer observed and the visual cortex is thought to become hardwired (Wiesel and Hubel, 1963a,b). On the other hand, the adult motor and prefrontal cortices retain a high level of plasticity throughout the lifespan, as seen for example in motor learning (Karni et al., 1995). This disparity in the plastic potentials of different brain regions in adulthood led to a modular approach to the study of neuroplasticity assuming different brain regions have their own plasticity mechanisms that do not depend or translate on others.

Growing evidence shows that the adult human visual cortex retains a particular form of OD plasticity: after a few hours of monocular deprivation, the deprived eye unexpectedly increases its dominance over the nondeprived eye, as measured by binocular rivalry (Lunghi et al., 2011, 2013, 2015a,b) and other tasks (Zhou et al., 2013, 2017; Bai et al., 2017; Wang et al., 2020). This counterintuitive OD shift also occurs at the neural level: short-term MD boosts deprived-eye related activity in the early visual cortex, while reducing responses to the nondeprived eye (Zhou et al., 2013, 2015; Lunghi et al., 2015a; Binda et al., 2017, 2018; Chadnova et al., 2017). Moreover, this effect is accompanied by a decrease in resting GABA concentration in the visual cortex (Lunghi et al., 2015b), suggesting the involvement of a homeostatic mechanism that aims preservation of a constant excitation/inhibition (E/I) balance (Turrigiano and Nelson, 2004). Interestingly, motor learning is also closely tied to GABA modulation in the motor cortex (Bütefisch et al., 2000). Decreased GABA concentration in the motor and sensorimotor cortices facilitates motor learning (Ziemann et al., 2001; Floyer-Lea et al., 2006) and individual variations in motor learning performance correlate with the changes in motor cortex GABA concentration following transcranial direct current stimulation (Stagg et al., 2011). Motor learning procedures such as sequence learning may require working memory resources too, which in turn involve changes in the E/I balance in the prefrontal cortex (Barak and Tsodyks, 2014), with a potential role for GABAergic inhibition (Bañuelos et al., 2014; Bañuelos and Wołoszynowska-Fraser, 2017; Hoskison et al., 2009; Rodermund et al., 2020).

That the adult visual system retains a degree of homeostatic plasticity higher than previously thought and that visual, motor plasticity and working memory share common neural mechanisms (E/I balance alteration) suggest that these forms of plasticity might be linked and controlled jointly to ensure overall stability in the brain. However, this hypothesis has never been tested directly. Here, we address this issue by investigating the interaction between visual and motor plasticity in adult humans using two well-established experimental paradigms to elicit each form of plasticity (i.e., short-term monocular deprivation (Lunghi et al., 2011; J. Zhou et al., 2013) and motor sequence learning (Seitz et al., 1990; Jenkins et al., 1994; Karni et al., 1995; Ungerleider et al., 2002; Hotermans et al., 2006). We designed a combined task in which visual and motor plasticity are induced at the same time and compared visual and motor plasticity in this condition to simple tasks in which either form of plasticity was induced on its own. Our results show a unilateral interaction between visual and motor plasticity that impairs visual plasticity in the presence of active motor plasticity. We further tested the contribution of working memory to this result by inducing visual plasticity at the same time as a working memory task without a motor learning component. We again found a unilateral interaction that impairs visual plasticity.

## Materials and Methods

### Human participants

34 participants (mean age 28.3 ± 4.8 years, 12 males) with normal or corrected-to normal visual acuity (measured with ETDRS charts) took part in the study. One subject was excluded from the analysis because of inconsistent ocular dominance index (ODI) within and across the binocular rivalry blocks on the same day, two others were excluded because of high variation in baseline ocular dominance on different days. Data from the remaining 31 participants (mean age 27.8 ± 4.7 years, 11 males) was analyzed. All subjects but two (the authors) were naive to the purpose of the experiment.

A subset of participants (*N* = 10, mean age 27.3 ± 3.6 years, 5 males) including the authors were included in the visual control study.

For the working memory control experiment we included three of the participants in the main cohort (including the authors) and recruited 18 more naive participants for a total of 21 participants (mean age 29.6 ± 6.3, 9 males).

### Ethics statement

The experimental protocol was approved by the local ethics committee (Comité d’éthique de la Recherche de l’université Paris Descartes, CER-PD:2019-16-LUNGHI) and was performed in accordance with the Declaration of Helsinki (DoH-Oct2008). All participants gave written informed consent. The naive participants received financial compensation of 10€ per hour.

### Apparatus and stimuli

#### Visual task: binocular rivalry

The experiment took place in a dark and quiet room. The visual stimuli were generated in MATLAB (R2020b, The MathWorks Inc.) using Psychtoolbox-3 (Brainard, 1997) running on a PC (Alienware Aurora R8, Alienware Corporation) and a NVIDIA graphics card (GeForce RTX2080, Nvidia Corporation).

Visual stimuli were presented dichoptically through a custom-built mirror stereoscope and each subject’s head was stabilized with a forehead and chin rest positioned 57 cm from the screen. Visual stimuli were two sinusoidal gratings oriented either 45° clockwise or counterclockwise (size: 3.1°, spatial frequency: 2 cpd, contrast: 50%), presented on a uniform gray background (luminance: 110 cd/m^2^, CIE *x* = 0.305, *y* = 0.332) in central vision with a central white fixation point and a common squared white frame to facilitate dichoptic fusion. The stimuli were displayed on an LCD monitor (BenQ XL2420Z 1920 × 1080 pixels, 144 Hz refresh rate). Responses were recorded through the computer keyboard. Monocular deprivation was performed using eye-patching. The eye-patch was made of a translucent plastic material that allowed light to reach the retina but prevented pattern vision, in accordance with previous studies (Lunghi et al., 2011, 2015a, 2019a).

#### Motor task: motor sequence learning

The motor learning experiment took place in a quiet and lit room. The experiment ran on a PC (Dell) with MATLAB using Psychtoolbox-3. A series of numbers in white were presented on a uniform gray background (luminance: 47.6 cd/m^2^, CIE *x* = 0.306, *y* = 0.335). Observers viewed the display at a distance of 50 cm on an LCD monitor (Dell 2016H, 1600 × 900 pixels, 60 Hz refresh rate, Dell Inc.). Responses were recorded through a four-buttoned response pad (four-button USB response pad carbon fiber effect, The Blackbox Toolkit Ltd.).

#### Visual control: story reading task

The experiment took place in a quiet and lit room. The experiment ran on a PC (Dell) with MATLAB using Psychtoolbox-3. Stories were displayed maximum two lines at a time in the center of the LCD monitor (Dell 2016H, 1600 × 900 pixels, 60 Hz refresh rate, Dell Inc.). The story lines were displayed in white against a uniform gray background (luminance: 47.6 cd/m^2^, CIE *x* = 0.306, *y* = 0.335). Participants used the arrow keys to navigate the stories.

#### Working memory control: working memory task

The experiment used the same materials and methods as in the simple visual and simple motor conditions. The stimuli for the simple visual and motor tasks were as described above. The working memory task ran on the same computer and monitor as in the simple motor task. Participants saw a series of white letters briefly flashed in the center on a uniform gray background (luminance: 44.8 cd/m^2^, CIE *x* = 0.299, *y* = 0.340). All participants observed the display from a 50 cm distance, stabilized by a chin rest, in a quiet and lit room. Responses were recorded through the computer keyboard.

### Procedures

All participants performed three different conditions: simple motor, simple visual and combined. The order of conditions was counterbalanced across subjects and each condition was performed on separate days. Because of recent evidence showing an interaction between visual homeostatic plasticity and energy metabolism (Kim et al., 2017), each condition was performed at approximately the same time of the day and immediately after lunch.

#### Simple visual task

**Figure 1.**
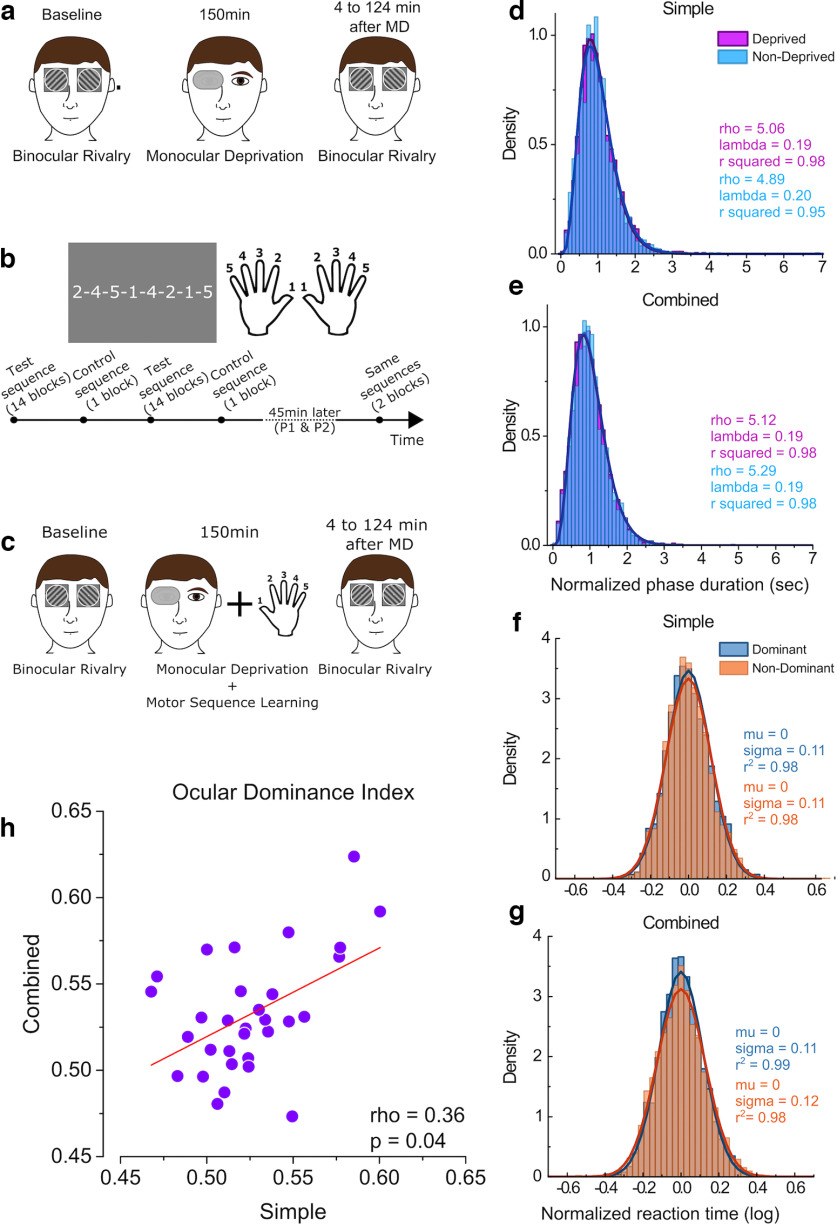
Experimental paradigm and response distributions. ***a***, In the simple visual condition, participants performed a binocular rivalry task before and after 150 min of monocular deprivation. Participants indicated the orientation of the grating they perceive. ***b***, In the simple motor task, participants were instructed to tap their fingers in the order displayed on the screen. Numbers corresponded to fingers. Each hand was trained separately. For each hand, participants practiced four different sequences: two test sequences (14 blocks) and two control sequences (1 block). Forty-five minutes after the initial training of a hand, participants performed the same four sequences for two blocks each. ***c***, In the combined condition, participants performed the motor task during the monocular deprivation (MD) period. Binocular rivalry was performed before and after the MD period. ***d***, ***e***, Normalized phase duration distributions in de baseline blocks of the simple and combined visual conditions. The distributions are fitted with a γ function (Eq. 1). ***f***, ***g***, Log-transformed reaction time distributions, normalized across participants, in the first block of simple and combined motor conditions. The distributions are fitted with a Gaussian function (Eq. 3). ***h***, Correlation between the ODI measured at baseline in the simple versus combined condition (performed on different days).

Ocular dominance was measured by means of binocular rivalry (Levelt, 1966; Alais et al., 2005). Visual plasticity was quantified as the ocular dominance shift occurring for each participant after short-term (150 min) monocular deprivation (Fig. 1*a*). Participants viewed a screen through a mirror stereoscope and reported the change in their perception via the keyboard by pressing one of three arrow keys continuously according to the orientation of the grating they perceived at the moment (left arrow: counterclockwise, right arrow: clockwise, down arrow: mixed). Each binocular rivalry block lasted 8 min including two 3-min trials with a pause in between. The 3-min trials consisted of two subtrials of 90 s and a 3-s break in the middle. Participants remained in position through a first trial, a 2-min break and a second trial of rivalry. After each break, grating locations were switched to avoid adaptation. The starting locations of the gratings were counterbalanced across participants.

After a baseline block of binocular rivalry, the participants wore an eye-patch for 150 min over their dominant eye. We used as operational definition of dominant eye the eye that dominated perception in binocular rivalry during the baseline block. When the ocular dominance in the baseline block was inconclusive (0.49 < ODI < 0.51, *N* = 9), we used the PORTA test (Lederer, 1961) where participants were asked to align their hands at arm’s length with an object in distance. They were then instructed to close one eye alternately. The eye that led to the most displacement in the alignment when closed was noted as the dominant eye. During the 150 min of monocular deprivation, participants were free to perform different activities in the lab like work, read or browse the web or even go for a walk outside. At the end of this period the eye-patch was removed and the participants performed seven more blocks of binocular rivalry. The first five blocks were separated by 7 min, whereas the last three blocks had 22 min in between. In total, ocular dominance was measured for 128 min following eye-patch removal. We calculated a unique ocular dominance index (ODI; see Eq. 2) for each of the eight blocks. The ocular dominance index difference from the baseline at each time point served as a measure of visual plasticity.

#### Simple motor task

A motor sequence learning task was used to assess motor plasticity. Motor plasticity for each participant was quantified as the decrease in reaction time (RT) after 14 blocks of practice. Participants were instructed to tap their fingers in the order of the eight-element number sequence shown on the monitor. Each number on the screen corresponded to a finger and the numbering was mirrored, that is, the thumb was always number 1 regardless of the hand (Fig. 1*b*). Our response pad having only four buttons, the number three was never shown on the screen so the middle finger was never used. Half of the participants started the training with their dominant hand and the other half with their nondominant hand. The training hand was occluded from their view by a cardboard box. Participants trained on four different finger tap sequences per hand (eight in total): two “test sequences” (repeated for 14 blocks) and two “control sequences” (repeated for only one block). The labeling and the order of these sequences were counterbalanced across participants.

The motor learning task consisted in a total of 30 blocks (14 × 2 test sequences and 1 × 2 control sequences) per hand. Each block consisted of five trials and each trial consisted of three subtrials each corresponding to a full repetition of a finger tap sequence. Participants were instructed to tap their fingers in the displayed order, as rapidly and as correctly as possible. When the training of all four sequences were complete for a hand, participants took a break before starting the training of the other hand. Approximately 45 min after the training of a hand, participants repeated all four sequences for two more blocks each to make sure the reaction time (RT) decrease was stable and specific to test sequences. The whole task lasted ∼150 min.

Reaction times (time elapsed from the first until the last finger tap in a subtrial) and accuracy were computed per each participant and for each experimental block. The reaction time decrease for test sequences from the first to the 14th block served as a measure of motor plasticity.

#### Combined task

In the combined condition, the simple visual and the motor tasks were performed at the same time (Fig. 1*c*). Participants started with the baseline block of binocular rivalry. After the baseline block, participants wore the eye-patch. Here, instead of engaging in their preferred activity, all participants performed the motor learning task during the monocular deprivation period. This way, we induced motor and visual plasticity simultaneously. At the end of the monocular deprivation and the motor task, we removed the eye-patch and the participants completed seven more binocular rivalry blocks. The same measures (RT, accuracy, and ODI) were recorded.

#### Visual control task

In order to eliminate the possibility of a confound in our study, we included a fourth (control) condition. Ten participants from the main experiment cohort of observers took part in the visual control condition. In this control study, the effect of short-term monocular deprivation was measured as in the simple visual task. However, during the 150 min of monocular deprivation, participants read stories (white text presented on a uniform gray background), to match the visual stimulation received during the combined task. We used the same screen and background as in the motor task and the stories were displayed maximum two lines at a time.

#### Working memory control task

To control for the possible effect of working memory load on monocular deprivation induced ocular dominance shift, we included another control condition in our study. Twenty-one participants took part in this experiment where we repeated the simple visual, combined visuo-motor plasticity and combined visual plasticity and working memory conditions. The effect of short-term monocular deprivation was measured as in the simple visual task. Participants performed a working memory task while they were under monocular deprivation. A set of three, five or seven letters were flashed sequentially and briefly (0.15 s each) on a gray screen with an inter stimulus interval of 1.5 s. Participants had a 3-s memory retention period at the end of the set and before the flashing of the probe letter. The probe letter was equally likely to be within or outside of the set on each trial. Participants indicated whether the flashed probe letter belonged to the set (left arrow key) or not (right arrow key). The response was followed by an auditory feedback indicating correct (high pitch) or incorrect (low pitch) response. Each block consisted in three trials containing 30 subtrials each. One block lasted about 15 min. Participants started the first block of the working memory task at 15 min into the monocular deprivation period. In total, they completed four blocks of working memory task with 20-min breaks in between. The starting condition (simple visual, simple motor or working memory control) was counterbalanced across participants.

### Statistical analyses

The recorded reaction times and perceptual durations were first analyzed on MATLAB and then the statistical tests were performed with SPSS statistics (version 25, IBM Corporation). The mean reaction times and ODI based on perceptual durations were separately compared with repeated measures ANOVAs. Although both the initial distribution of our data and the residuals of the ANOVAs significantly differed from normality, literature has shown that ANOVAs are quite robust in Type I and II errors and power with non-normal distributions (Harwell et al., 1992; Schmider et al., 2010) and are arguably preferable to alternatives (Knief and Forstmeier, 2021). Pairwise comparisons are made with Wilcoxon signed rank tests. Multiple comparisons were corrected with Holm–Bonferroni method.

#### Visual task

For the analysis of the binocular rivalry data, we discarded phase durations <0.25 s to eliminate keypress errors. To plot perceptual phase duration distributions for the deprived and nondeprived eyes in the baseline blocks of the two different conditions, we first normalized each participant’s phase duration to their own mean. The distributions of the pooled data of all participants then were fitted by a two-parameter γ distribution (r, λ) of the form:

(1) $$g\left(x\right)=\frac{\lambda^{r}x^{r-1}}{\text{Γ}\left(r\right)}e\left(-\lambda x\right),$$

where Γ is the *γ function*, *r* is the shape parameter and *λ* is the scale parameter. The goodness of fit (*r*^2^) was >0.97 for all distributions (Fig. 1*d*,*e*).

We compared the ocular dominance index change in the two conditions (simple vs combined). We calculated the ocular dominance index (ODI) as follows:

(2) $$ODI=\frac{{Time}_{DE}}{{Time}_{DE}+{Time}_{NDE}}.$$

Where Time_DE_ indicates the total amount of time (in seconds) spent by the observer reporting the dominance of the stimulus presented to the deprived eye and Time_NDE_ indicates the total amount of time (in seconds) spent by the observer reporting the dominance of the stimulus presented to the nondeprived eye. ODI values around 0.5 indicate, therefore, balanced ocular dominance, while ODI > 0.5 indicates predominance of the deprived eye and ODI < 0.5 predominance of the nondeprived eye. We excluded the participants whose baseline ODI on the two days showed a difference larger than 0.12. The reason for this exclusion criterion is that the effect of monocular deprivation on the ocular dominance change is estimated to be about a 0.12 change in ODI based on the literature (Lunghi et al., 2019a). Data from participants who show a natural variation larger than this value would not reflect the effect of monocular deprivation.

We subtracted the baseline ODI of each participant from the subsequent block ODIs to see the effect of patching on ocular dominance. We then compared the ODI change in two conditions (simple vs combined) in a 7 (Blocks) × 2 (Conditions) repeated-measures ANOVA. The binocular rivalry blocks taking place after the monocular deprivation period were binned: 0–8, 15–23, 30–38, 45–53, 60–68, 90–98, and 120–128 min.

#### Motor task

In the analysis of reaction times (RT), only the correct trials were taken into account (incorrect trials 13.7% for simple motor and 13.9% for combined condition). Outlier RTs (defined as the RTs mean ± 4 SD of each participant) were removed (0.3% each for both simple motor and combined conditions). We looked at the reaction time distributions for dominant and nondominant hands within the first block of each condition by first dividing each participant’s data to their mean RT. The distributions based on these raw reaction times showed very high skewness and kurtosis. To work around this, the reaction times were log-transformed and normalized by subtracting the mean of each participant from their data. The pooled reaction time distributions were then fitted by a two-parameter Gaussian distribution (µ, σ) of the form:

(3) $$f\left(x\right)=\frac{1}{\text{σ}\sqrt{2\pi }}e^{-\frac{1}{2}{\left(\frac{x-\mu }{\sigma }\right)}^{2}}.$$

Where f is the probability density function, µ is the mean and σ  is the SD. The goodness of fit (*r*^2^) was >0.98 for all distributions (Fig. 1*f*,*g*).

Working with the log-transformed reaction times, we calculated each participant’s mean reaction time in each block. We then subtracted each participant’s mean reaction time in the first block from all others, equalizing all participants to a starting point of 0.

We compared the mean reaction time differences from the first to the 14th block for only test sequences (dominant and nondominant hand together) across the two conditions (simple vs combined) using a 13 (Blocks) × 2 (Conditions) repeated-measures ANOVA.

#### Correlation

To check for correlation between visual and motor plasticity, we used the data from simple visual and simple motor tasks. The visual plasticity score for each participant was calculated by subtracting the baseline ODI from the ODI immediately after monocular deprivation (0–8 min). The motor plasticity score for each participant was calculated by subtracting the mean RT difference from baseline on the 14th or the second block from 0. Correlations between visual and motor plasticity scores were computed using the Spearman’s rank correlation coefficient (ρ) and *p*-values by using the permutation distributions.

## Results

We assessed visual homeostatic plasticity and motor plasticity in a group (*N* = 31) of adult volunteers using two well established experimental paradigms. We quantified visual homeostatic plasticity as the shift in ocular dominance (measured by binocular rivalry) occurring after 150 min of monocular deprivation (simple visual task; Fig. 1*a*). The ocular dominance index (ODI; see Eq. 2) measured before deprivation (baseline ODI) in the simple and combined conditions correlated across participants (ρ = 0.36, *p* = 0.04; Fig. 1*h*), indicating that the ODI is a reliable measure of ocular dominance. Across the two experimental sessions we also found strong correlations for two other binocular rivalry parameters: mean phase durations (ρ = 0.80, *p* = 5.3e-7; data not shown) and mixed percept proportions (ρ = 0.74, *p* = 3.9e-6; data not shown). We assessed motor plasticity as the reaction time decrease over fourteen blocks of finger-tap-sequence learning (simple motor task; Fig. 1*b*). In a third condition, visual and motor plasticity were elicited at the same time, as participants performed the motor task during the 150 min of monocular deprivation (combined task; Fig. 1*c*). Phase duration distributions in binocular rivalry and reaction time distributions for the motor learning task in the different experimental conditions are reported in Figure 1*d–g*. The phase duration distributions for binocular rivalry follow a γ distribution (*r*^2^ > 0.97) as expected (Levelt, 1966). The log-transformed reaction time distributions are well fit by normal distribution (*r*^2^ > 0.98).

**Figure 2.**
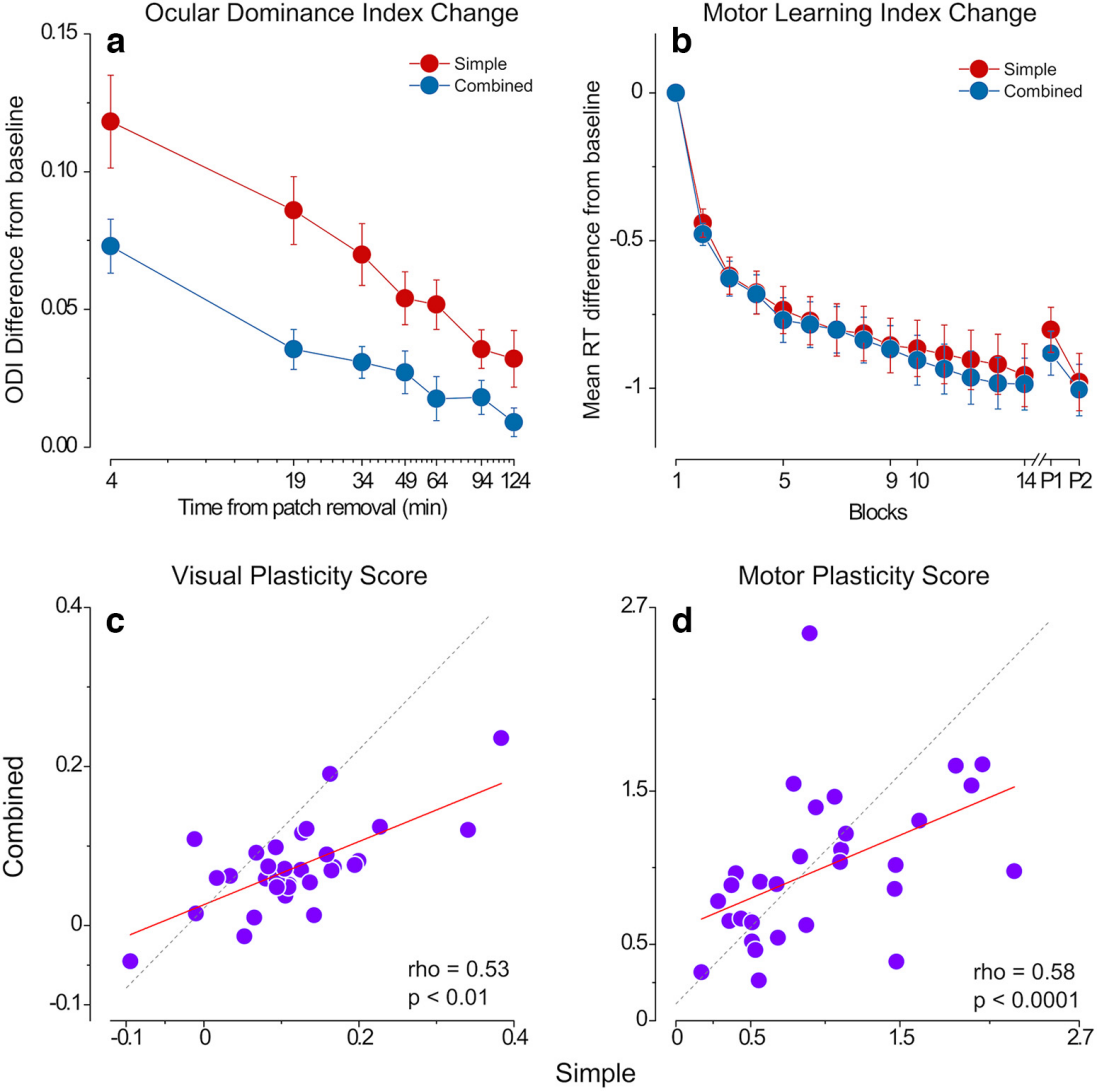
Results. ***a***, Ocular dominance index (ODI) difference from baseline for the simple visual (red symbols) and combined (blue symbols) conditions. Error bars represent 1 ± SEM. ***b***, Change in reaction times (RT) during motor learning (difference from baseline mean RT) in the simple (red symbols) and combined (blue symbols) conditions. Error bars represent 1 ± SEM. ***c***, Scatter plot of the ODI difference measured during the first 8 min after monocular deprivation in the simple versus combined condition. ***d***, Scatter plot of the RT change from baseline in 14th block in the simple versus combined condition.

Results from the simple visual and motor task replicate the effects established in the literature (Karni et al., 1995; Lunghi et al., 2011). Ocular dominance shifts in favor of the deprived eye after monocular deprivation (Wilcoxon signed ranks *z* = 4.56, *p* = 5e-06) and slowly returns to baseline after patch removal (Fig. 2*a*, red symbols). Reaction times consistently decrease over fourteen blocks of learning for the motor task [Wilcoxon signed ranks *z* = 4.85, *p* = 1.2e-06 (corrected: 4.7e-06)] and this decrease is maintained 45 min after initial training [Wilcoxon signed ranks *z* = 4.85, *p* = 1.2e-06 (corrected: 4.7e-06); Fig. 2*b*, red symbols]. The reaction times of test sequences remained significantly lower than those of control sequences 45 min after training [P1: Wilcoxon signed rank *z* = −3.1, *p* = 0.0017 (corrected: 0.0017), P2: Wilcoxon signed rank *z* = −3.7, *p* = 2e-04 (corrected: 0.0004)].

**Figure 3.**
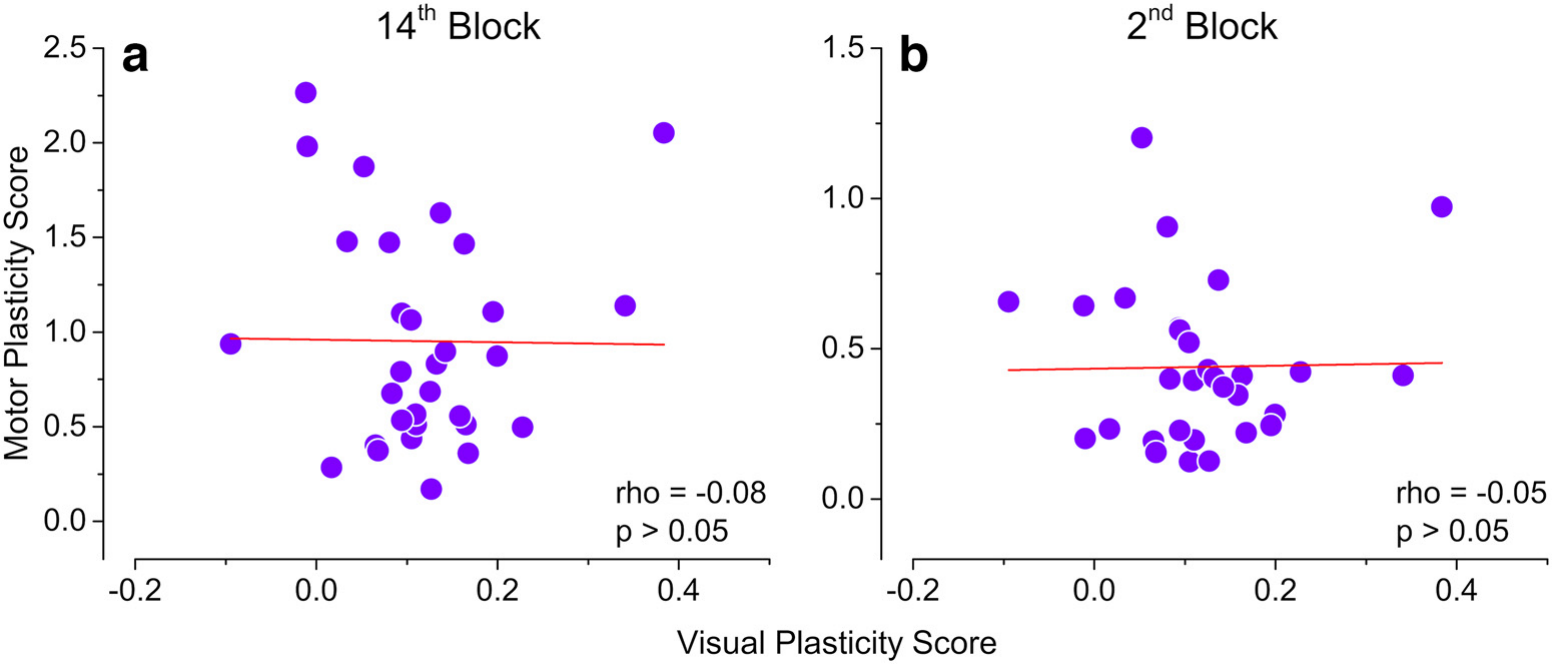
Correlation between visual and motor plasticity. ***a***, Correlation between the visual plasticity score (ODI change in 8 min-after-deprivation) and motor plasticity score based on mean reaction time difference from baseline in the last block (0 - mean RT difference in 14th block). ***b***, Correlation between the visual plasticity score (same as panel ***a***) and motor plasticity score based on reaction time difference from baseline in the second block (1, mean RT difference in second block).

Interestingly, in the combined condition, we observed a significantly smaller shift in ocular dominance following short-term monocular deprivation compared with the simple visual task (Fig. 2*a*, blue symbols), indicating that eliciting visual and motor plasticity at the same time impaired the visual homeostatic response to monocular deprivation. A two-way (Block × Condition) repeated measures ANOVA showed a significant main effect of both the factor Block (*F*_(6,180)_ = 27.9, *p* = 1.8e-19, η^2^p = 0.48) and Condition (*F*_(1,30)_ = 14.4, *p* = 0.001, η^2^p = 0.32) and a significant interaction between the two (*F*_(6,180)_ = 2.76, *p* = 0.013, η^2^p = 0.08). The ODI change in the block immediately after monocular deprivation (i.e., visual plasticity score) was correlated across the two conditions (ρ = 0.53, *p* = 0.0025), although the change in the simple condition was larger for most of the participants (Fig. 2*c*), indicating a good test-retest reliability of binocular rivalry in quantifying the effect of short-term monocular deprivation. No significant difference in motor learning was observed in the combined condition compared with the simple task (Fig. 2*b*, blue symbols): a two-way (Block × Condition) repeated measures ANOVA revealed a significant main effect of the factor Block (*F*_(12,360)_ = 49.9, *p* = 3.5e-69, η^2^p = 0.62), whereas the factor Condition (*F*_(1,30)_ = 0. 01, *p* = 0.923, η^2^p = 0.0) and the interaction of the two (*F*_(12,360)_ = 0.44, *p* = 0.944, η^2^p = 0.01) remained insignificant. The mean reaction time difference from the baseline on the 14th block (i.e., motor plasticity score) correlates across the two conditions (ρ = 0.56, *p* = 0.0013; Fig. 2*d*). In both conditions, participants performed the motor task with high accuracy rates throughout the training (mean = 0.87, SD = 0.10). We found no significant difference between the reaction times of the dominant and the nondominant hands nor a difference between the first-trained versus second-trained hands or sequences. When visual and motor plasticity are elicited simultaneously, motor plasticity is not impacted.

Having shown that visual and motor plasticity interact with each other, we investigated whether a correlation exists between the plastic potentials of the visual and motor cortex. We found no correlation between visual and motor plasticity across participants. We calculated two plasticity scores, one representing the maximal effect of motor learning (difference of the mean reaction time on the last block from the first block of training) and one indicating the speed of learning (difference in mean reaction times from the first to the second block). Neither of these motor plasticity scores correlates with the visual plasticity score [ρ = −0.08, *p* = 0.657 (Fig. 3*a*), ρ = −0.05, *p* = 0.749 (Fig. 3*b*)].

**Figure 4.**
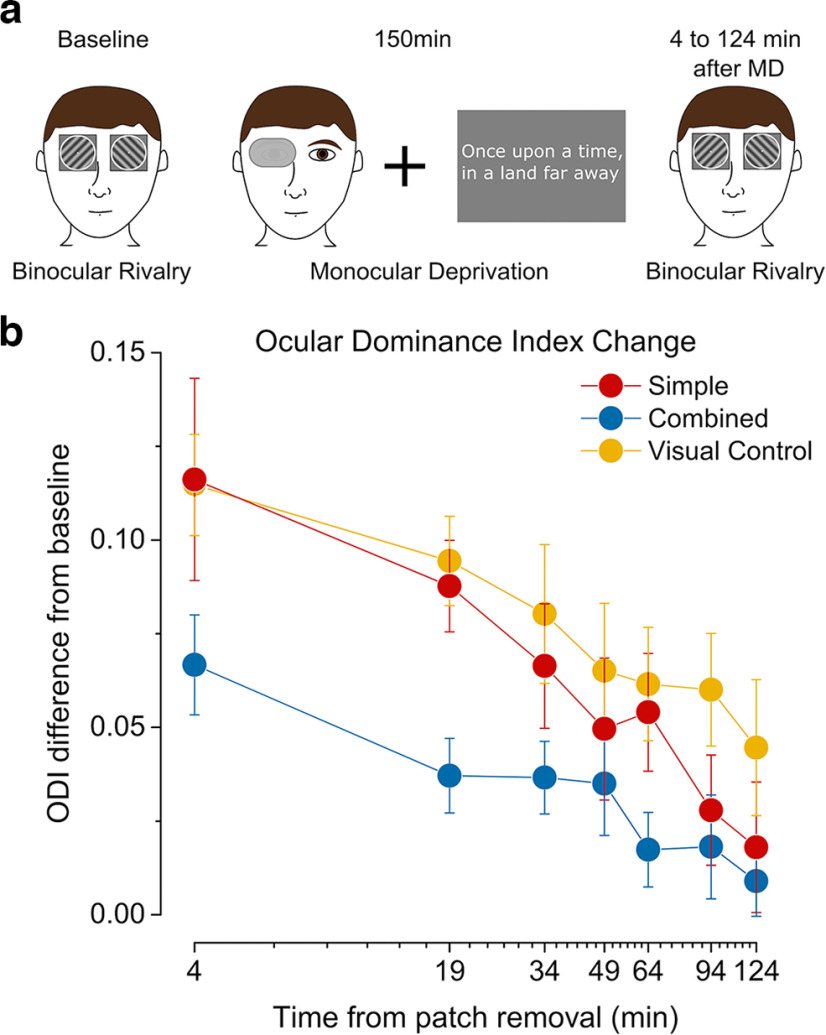
ODI change in the control task. ***a***, In the control study, during the monocular deprivation, 10 participants read short stories on a gray background, displayed a few lines at a time. ***b***, ODI change after monocular deprivation for the simple visual (red symbols), combined (blue symbols), and control (yellow symbols) condition. Error bars represent 1 ± SEM.

One possible confound, which could potentially affect the ocular dominance shift induced by monocular deprivation, is the difference in visual stimulation during monocular deprivation for the simple visual task and the combined task as shown by a previous study (Kim et al., 2017). While in the simple visual task, during monocular deprivation participants were free to perform their preferred activities and had therefore access to a rich visual environment, in the combined condition, visual stimulation during deprivation was reduced to the finger-tap sequence numbers displayed on a gray background. In order to address this possible confound, we performed a control experiment in which we measured the effect of 150 min of monocular deprivation on 10 participants who also performed the main experiment. In this visual control condition, during monocular deprivation, participants read stories displayed on a gray background reproducing the same amount of visual stimulation as in the combined condition (Fig. 4*a*). We found that the shift in ocular dominance observed in the control condition was comparable to that observed in the simple visual task (Fig. 4*b*). A two-way ANOVA (Block × Condition) confirmed that while in both conditions the ocular dominance index changed with blocks (*F*_(6,54)_ = 13.6, *p* = 2.2164e-9, η^2^p = 0.60), there was no significant effect of condition (*F*_(1,9)_ = 1.59, *p* = 0.239, η^2^p = 0.15), nor interaction between the two (*F*_(6,54)_ = 0.94, *p* = 0.47, η^2^p = 0.09). A two-way (Block × Condition) repeated measures ANOVA between control and combined conditions showed a significant main effect of both the factor Block (*F*_(6,54)_ = 9.78, *p* = 2.7792e-7, η^2^p = 0.52) and Condition (*F*_(1,9)_ = 9.55, *p* = 0.013, η^2^p = 0.51) while the interaction between the two (*F*_(6,54)_ = 0.59, *p* = 0.73, η^2^p = 0.06) remained insignificant. This indicates that the drop in the monocular deprivation effect observed in the combined condition was not because of poor visual stimulation.

**Figure 5.**
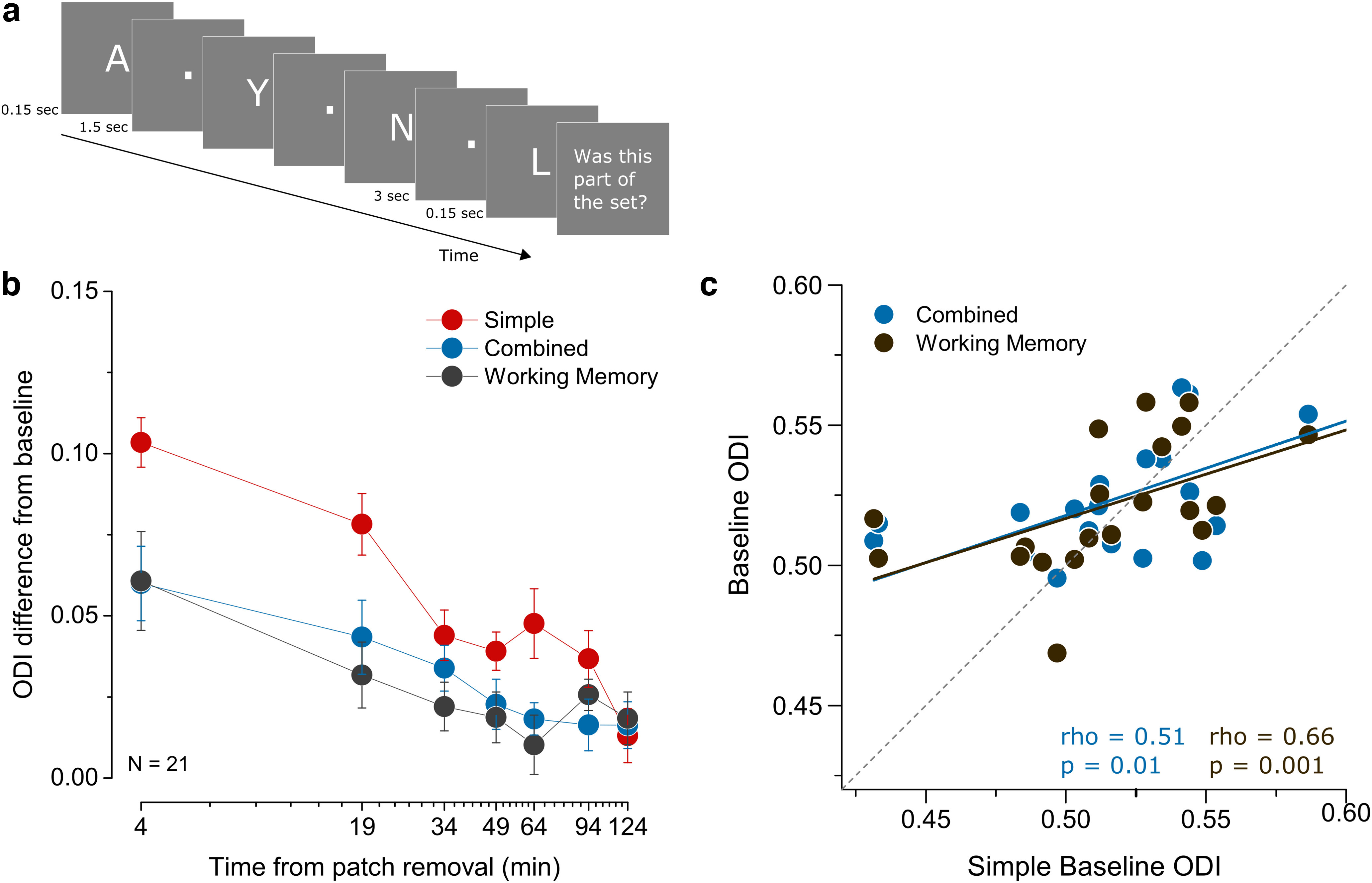
Working memory task. ***a***, In the working memory study, 21 participants performed a working memory task during the monocular deprivation. A set of white letters were briefly flashed on a gray background. After a retention period of 3 s, participants saw a probe letter flashed and judged whether it was part of the set. They received an auditory feedback after their response. ***b***, ODI change after monocular deprivation for the simple visual (red symbols), combined (blue symbols) and working memory control (black symbols) condition. Error bars represent 1 ± SEM. ***c***, Correlation of baseline ocular dominance index in the repeated simple condition and the working memory and combined conditions.

Another potential contributor to our results was the discrepancy in the working memory load between the simple visual task and the combined task during monocular deprivation. Although there is no clear evidence for a likely impact of working memory on the ocular dominance shift induced by short-term monocular deprivation, the motor sequence learning task nevertheless uses resources related to both motor learning and working memory. To understand the contribution of working memory engagement to the observed effect, we performed another control experiment in which, during monocular deprivation, the participants performed a working memory task reproducing the demand on working memory as in the simple motor task (Fig. 5*a*). All participants of this working memory control condition also repeated the simple visual condition and the combined condition. In baseline measurements, the ODI was strongly correlated for the three conditions (Simple-Combined ρ = 0.51, *p* = 0.01 and Simple-Working Memory ρ = 0.66, *p* = 0.001; Fig. 5*c*), indicating good test-retest reliability. A two-way ANOVA (Block × Condition) between the simple visual and combined conditions once again showed significant effects for block (*F*_(6,120)_ = 20.9, *p* = 1e-16, η^2^p = 0.51), Condition (*F*_(1,20)_ = 7.18, *p* = 0.014, η^2^p = 0.26), and their interaction (*F*_(6,120)_ = 3.89, *p* = 0.001, η^2^p = 0.16), replicating the main result (Fig. 5*b*). On the other hand, we found an effect of working memory on the ocular dominance shift (Fig. 5*b*). A two-way ANOVA (Block × Condition) between the simple visual and working memory conditions showed an effect of block (*F*_(6,120)_ = 20.8, *p* = 1.2e-16, η^2^p = 0.51), as expected. Here, we also observed an effect of condition (*F*_(1,20)_ = 6.73, *p* = 0.017, η^2^p = 0.25), indicating a difference in the ocular dominance shift in the two conditions and a significant interaction of the two factors (*F*_(6,120)_ = 5.39, *p* = 5.8e-5, η^2^p = 0.21).

## Discussion

Neuroplasticity in the adult brain has been canonically studied in a modular fashion, supporting the view that plasticity acts locally in different brain areas. Here, we investigated for the first time the interaction between two different forms of plasticity by inducing visual and motor plasticity either separately or at the same time in a group of adult human volunteers. We found a unilateral interaction between these two forms of plasticity: when visual and motor plasticity are induced at the same time, visual plasticity is drastically reduced, and this reduction is not attributable to poorer visual stimulation. In another control experiment, we found that visual homeostatic plasticity is also reduced when a visual working memory task is simultaneously performed. Taken together, our results suggest that neuroplasticity is not fully confined to modules in the brain but is subject to interactions, likely to maintain overall homeostasis and prevent excessive instability.

One of the neural mechanisms underlying different forms of plasticity is the balance between excitation and inhibition (E/I), with a predominant role of GABAergic inhibition. Studies from both animal models and humans have shown that decreasing intracortical GABA levels promote visual (Harauzov et al., 2010; Greifzu et al., 2014), motor (Bütefisch et al., 2000; Ziemann et al., 2001), and hippocampal (McLean et al., 1996; Paulsen and Moser, 1998) plasticity. Indeed, it has been shown that GABAergic inhibition is also involved in the two paradigms that we used to induce visual and motor plasticity: motor sequence learning induces a decrease of the GABA levels in the motor cortex (Floyer-Lea et al., 2006; Stagg et al., 2011; Kolasinski et al., 2019) and a reduction in visual cortical GABA concentration has been observed after short-term monocular deprivation (Lunghi et al., 2015b). It is therefore possible that the observed interaction between motor and visual plasticity is mediated by a disruption of the E/I balance. This hypothesis is consistent with evidence from animal models showing that a localized stroke in S1 or M2 abolishes ocular dominance plasticity (Greifzu et al., 2011; Pielecka-Fortuna et al., 2015). After a stroke, a reduction of GABA concentration occurs at the level of the motor and the sensorimotor cortex. This response enhances plasticity and therefore promotes recovery in the lesioned area (Manganotti et al., 2008; Kim et al., 2014; Blicher et al., 2015; Alia et al., 2016).

The changes in the E/I balance are also essential to working memory processes. While the mechanisms of working memory are still being debated, two main proposals both rely on changes in the E/I balance. Early studies looking into neural mechanisms of working memory found that the short-term retention of information is mediated by sustained activity in the prefrontal cortex (Fuster and Alexander, 1971; Funahashi et al., 1989; Chafee and Goldman-Rakic, 1998; Rainer et al., 1998; Romo et al., 1999). This sustained activity requires a shift in the E/I balance with a key role for fast inhibition (Goaillard et al., 2010; Boerlin et al., 2013; Lim and Goldman, 2013; Barak and Tsodyks, 2014). Although later studies have questioned the sustained activity hypothesis (Lundqvist et al., 2016, 2018a, b; Kucewicz et al., 2017; Bastos et al., 2018), the alternative explanation still relies on E/I balance changes. This alternative account proposes that information is stored in synaptic weights (Erickson et al., 2010; Trübutschek et al., 2017; Masse et al., 2019), relying on fast Hebbian plasticity (Sandberg et al., 2003; Fiebig and Lansner, 2017; Fiebig et al., 2020; Huang and Wei, 2021), short-term facilitation (Mongillo et al., 2008), or anti-Hebbian processes (Kozachkov et al., 2022) according to different neural models. While the main locus of working memory in the brain is thought to be the prefrontal cortex, according to sensory recruitment hypothesis, other sensory systems processing the relevant information also play a crucial role (Zhou and Fuster, 1996; Lorenc et al., 2018; Scimeca et al., 2018; Rademaker et al., 2019; Jia et al., 2021; Yu and Postle, 2021; Zhao et al., 2022; Phylactou et al., 2022; but for a reevaluation of the sensory recruitment hypothesis, see Masse et al., 2020). In our experiment, the visual working memory task that we implemented would lead to E/I changes in the prefrontal cortex but potentially in the visual cortex too even at levels as low as the V1 (Harrison and Tong, 2009; Serences et al., 2009; Christophel et al., 2012; Riggall and Postle, 2012; Rademaker et al., 2019; Jia et al., 2021; Zhao et al., 2022), possibly mediating the interaction that we observe with visual homeostatic plasticity. There is also some evidence pointing toward a role of GABAergic inhibition in working memory processes by linking GABA disruption to disturbed performance (Hoskison et al., 2009; Bañuelos et al., 2014; Rodermund et al., 2020) or taking GABA levels as predictors for performance (Michels et al., 2012; Yoon et al., 2016; Bañuelos and Wołoszynowska-Fraser, 2017). These common neural mechanisms between visual homeostatic plasticity and working memory might further contribute to the interaction between working memory and the ocular dominance shift.

We therefore speculate that the negative influence of motor learning or working memory activity on visual plasticity that we observed might be because of a global regulatory mechanism on plasticity. The disruption in E/I balance in the motor or the prefrontal cortex might limit the extent to which E/I can be altered in the visual cortex. The reason for this interruptive influence might be maintaining global homeostasis between excitation and inhibition in the brain to avoid extreme instability. When it comes to plasticity, the motor cortex might be taking priority and depressing the visual cortex since the visual environment is more stable and motor plasticity is more needed on a general basis. In a similar vein, prefrontal cortex might be taking precedence because of its necessity and relevance in the day-to-day life.

While the results on the interaction of visual and motor plasticity are put into question by the role of working memory, as it also seems to interrupt visual plastic processes, attributing the observed effect solely to working memory would be a premature conclusion. First, as presented above, there is a body of work supporting an interaction between motor and visual plasticity without the involvement of working memory (Greifzu et al., 2011; Pielecka-Fortuna et al., 2015). Second, the types of working memory used in the two tasks, i.e., motor sequence learning and working memory, remain non identical. While the motor sequence learning task would mainly require motor working memory, in the working memory condition the task we adopted is purely visual. Thus, the distinction between the influences of motor plasticity and working memory is yet to be made. It is likely that working memory contributed to the interaction effect between visual and motor plasticity observed in the combined condition. However, it remains possible that even without a working memory component, motor plasticity disrupts visual plasticity albeit in a less dramatic manner. Further studies looking into motor cortex plasticity and visual plasticity without the involvement of working memory are necessary to draw more concrete conclusions about the respective roles of these processes and to better understand the neural mechanisms behind. For instance, altering the E/I balance in the motor cortex by means of transcranial magnetic stimulation and looking into its effect on visual plasticity would clarify the nature of interaction between motor and visual plasticity without involving any role for working memory. Imaging studies measuring GABA concentration in different regions of the brain during the simultaneous activation of different forms of plasticity could also help understand the contribution of GABAergic inhibition to the observed interactions.

While our results show an interaction between motor and visual plasticity, we did not find a correlation between the two. Assuming the plastic capacities of different regions of the brain are linked, we expected to see a positive or negative correlation between the two plasticity scores. The correlation for working memory and the other two forms of plasticity is not assessed as the former was intended solely as a control condition. The lack of correlation in our results can be because of the different nature of the tasks we used. Motor sequence learning induces a Hebbian form of plasticity that is mainly similar to long-term potentiation (LTP-like) (Ziemann et al., 2004; Stefan et al., 2006; Rosenkranz et al., 2007). The ocular dominance shift observed after short-term monocular deprivation, on the other hand, is a result of homeostatic mechanisms (Lunghi et al., 2013, 2015a, b; Binda et al., 2017). Homeostatic plasticity is short-lived and aims to preserve a certain E/I balance in the cortex as can be seen with the return to baseline after patch removal. LTP-like plasticity, in motor learning, comes with long-lived changes and goes through a consolidation phase after the initial training. We speculate that, while the lack of correlation between visual and motor plasticity confirms that these two forms of plasticity act at the local level, the interaction between the two indicates the existence of a global homeostatic mechanism regulating local plasticity in separate systems.

Although we included a control for the role of visual stimulation, ruling out a main contribution of this factor in explaining the observed interaction between the three types of plasticity, our study lacked a control condition for the role of motor activity. In the combined condition, the participants continuously performed finger taps during the monocular deprivation whereas in the simple visual condition there was no structured motor activity. Despite the fact that the motor sequence learning task we chose does not impose a high level of motor activity on the participants, one might argue that the effect that we see is due simply to motor activity and is not specifically related to motor plasticity. We believe that this is unlikely because of two reasons: first, evidence from previous studies have shown that motor activity has either a boosting (Greifzu et al., 2014; Lunghi and Sale, 2015; Greifzu et al., 2016; Lunghi et al., 2019b) or a neutral (Finn et al., 2019) effect on visual plasticity. Second, in the simple visual condition, during monocular deprivation participants were free to perform motor activity: they all used their phones or computers, performing finger taps analogous to those involved in the motor learning task.

Taken together, our results hint at the existence of a global regulation of plasticity in the brain modulating the local expression of plasticity in separate systems. Alternatively, rather than being a systems-interaction, our results might be seen as evidence for interactions between different forms of plasticity, namely, homeostatic, Hebbian, and short-term synaptic plasticity. Our proposed mechanism behind these interactions remains the same. We speculate that changes in the motor or prefrontal cortex E/I balance mediated by GABA regulation have modulated the visual cortex E/I balance in the opposite direction to ensure overall stability of activity in the brain and preserve general homeostasis. Given the abundance of environmental conditions requiring motor adaptation or working memory engagement in contrast to the relative stability of the visual function, we propose that the motor and prefrontal cortex are less restricted in the disruption of E/I balance and hence can take priority over the visual cortex. Further studies focusing on the exact nature of these interactions are in order. Understanding the links between different forms of plasticity is the key for more efficient and innovative approaches to rehabilitative interventions on neurologic diseases and brain injury. The ability to tie together different forms of plasticity may prove useful to recovery from visual or motor dysfunction as seen by the advances in treatment of amblyopia (Lunghi et al., 2019b).

## Synthesis

Reviewing Editor: Ifat Levy, Yale University School of Medicine Decisions are customarily a result of the Reviewing Editor and the peer reviewers coming together and discussing their recommendations until a consensus is reached. When revisions are invited, a fact-based synthesis statement explaining their decision and outlining what is needed to prepare a revision will be listed below. The following reviewer(s) agreed to reveal their identity: Ella Striem-Amit.

The researchers performed a combination of motor learning and visual monocular deprivation experiments to probe the interaction when eliciting two types of plasticity at once. They discovered that as compared to both experiments performed separately, their combination did not affect the motor learning and plasticity, but did reduce the visual ocular dominance shift. The authors propose this shows a mechanism coordinating plasticity across different systems in the brain, to prevent excessive plasticity and instability. The authors performed two additional control experiments, one controlling for visual stimulation content and one controlling for attention and working memory, which differ between the simple presentation of each experiment separately and the joint experiment in the original experiment.

Overall, testing the interaction of plasticity across systems is novel and important, the design is simple yet elegant, and the results are interesting. The reviewers, however, had several comments that should be addressed, and require an additional experiment.

1. Reviewers appreciated the control experiments conducted, but were slightly alarmed at the results of the attentional control task, where the values of the ODI change for both simple and control conditions resemble more those of the combined condition in the main experiment than the simple visual condition. The authors speculate that this is due to different visual setups, but it would be more convincing to show that even under attentional control task there is a difference between this condition and the combined visual-motor plasticity condition. As it is, it’s hard to interpret the results of this important control.

2. Given that the outcomes of the visual experiment depend largely on the assumption of stability of the ocular dominance at baseline, can the authors provide these values across individuals, beyond those who were excluded based on this criterion? “The ocular dominance index measured before deprivation (baseline ODI) in the simple and combined conditions correlated across participants”, but the correlation isn’t very high (rho = 0.36, p = 0.04): how stable is it across two halves of the baseline condition in each condition?

3. The authors perform many post-hoc statistical tests, and it is not clear whether they are corrected for multiple comparisons. Correction will likely not affect the main findings, but should be done nonetheless.

4. It is important to state that any neural interpretation is rather speculative, as this design only includes behavioral experiments. Nevertheless, the discussion here is valid and well in line with previous literature. The authors should consider suggesting specific future experiments (maybe imaging- or spectroscopy-based) to study their suggested mechanisms.

Minor points:

1. The authors report that there was no effect of interference of previously learned motor sequences on new ones, as the same participants performed both simple motor experiment and combined visual-motor experiments. Can they show this data in the supplementary material?

2. Please report exact p-values rather than ranges (p> 0.05) for reproducibility.

3. Please provide more detail on how the PORTA test was used (line 138), and in how many participants ocular dominance in the baseline blocks was inconclusive in the baseline block of binocular rivalry.

4. The authors speculate that “the negative influence of motor plasticity on visual plasticity that we observed might be due to motor projections on the visual cortex” (line 409) - that’s an interesting statement. What is an anatomical pathway that would support this coordination?

5. The authors interpret the unilateral interaction between the two plasticity values studied here as motor plasticity overriding and interfering with visual plasticity. But as they also acknowledge, these are two types of plasticity: LTP-based and homeostatic plasticity. Could the interference direction be driven by the type of plasticity rather than the brain system (visual, motor) involved?

6. The fact that 2 participants (the authors) were not naïve to the purpose of the experiment seems rather unusual. While this is unlikely to have had a major effect on the results, it might be reassuring to check what would be the effect of the exclusion of these two.
